# Supplementary material for: Population structure and phenotypic variation of Sclerotinia sclerotiorum from dry bean (Phaseolus vulgaris) in the United States
Source: PeerJ. 2017 Dec 7;5:e4152. doi: 10.7717/peerj.4152 (PMC5723432; doi:10.7717/peerj.4152)
Supplement: Supplemental Information 1 [file peerj-05-4152-s001.pdf]

SUPPLEMENTARY INFORMATION

Table S1. Description of *Sclerotinia sclerotiorum* isolates used in this study. N = Number of Isolates. Key abbreviations: wmn = white mold screening nursery, producer = producer field, unk = unknown cultivar.

Country	State	Field Code	Year	Host	N
USA	CA	wmn	2004, 2005	Beryl, Bunsi, G122	18
USA	CO	producer	2007, 2010	Pinto, Yellow	41
		wmn	2003	GH	1
USA	ID	producer	2003	GH	1
USA	MI	wmn	2003, 2004, 2005, 2008, 2009	11A, 37, 38, B07104, Beryl, Bunsi, Cornell, G122, Orion, PO7863, WM31	43
		producer	2003, 2008, 2009	BL, Black, Fuji, GH, Merlot, SR06233, unk, Vista, Zorro	19
USA	MN	wmn	2003, 2004	Beryl, Bunsi, G122	11
USA	ND	producer	2007, 2010	unk	53
		wmn	2005	Beryl, Bunsi, G122	7
USA	NE	wmn	2004, 2005, 2008, 2010	Beryl, Bunsi, G122, PO7683, unk	27
		producer	2003, 2007, 2009, 2010	Beryl, Emerson, GH, Orion, Pinto, Weihing	20
USA	NY	producer	2003	GH	1
USA	OR	wmn	2003, 2004	Beryl, Bunsi, G122	15
		producer	2003	G122, GH	2
USA	WA	wmn	2003, 2004, 2005, 2008	11A, 37, 38, Beryl, Bunsi, Cornell, G122, Orion, PO7 104, PO7863, WM31	36
		producer	2003, 2007	GH, Merlot, Pinto, Redkid	23
USA	WI	producer	2003	GH	2
Mexico	-	wmn	2005	Beryl, Bunsi, G122	18
France	-	wmn	2004, 2005	Beryl, Bunsi, G122	18
		producer	2012	unk	4
Australia	-	wmn	2004	Beryl, Bunsi, G122	4
		producer	2004	Beryl	2

Table S2. Mean aggressiveness ratings for Regions with more than five samples; groupings according to 95% family-wise confidence interval.

Region	Mean Aggressiveness	Group
MN	5.84	a
ND	5.77	a
NE	5.29	ab
MI	5.13	abc
OR	4.84	abcd
CO	4.72	bcd
WA	4.67	cd
France	4.66	cd
Mexico	4.58	cd
Australia	4.12	cd
CA	4.01	d

Table S3. Mean aggressiveness ratings for the 10 most abundant MCG; groupings according to 95% family-wise confidence interval.

MCG	Mean Aggressiveness	Group
44	6.03	a
3	5.50	ab
5	5.40	b
2	5.25	b
9	5.11	b
1	4.95	b
45	4.88	b
4	4.87	b
53	4.69	b
49	4.60	b

Table S4. Mean aggressiveness ratings for the 10 MLH most abundant; groupings according to 95% family-wise confidence interval.

MLH	Mean Aggressiveness	Group
78	6.07	a
65	5.94	a
9	5.67	ab
25	5.41	ab
66	5.30	ab
104	5.22	ab
160	4.80	ab
163	4.80	ab
165	4.34	b
140	4.31	b

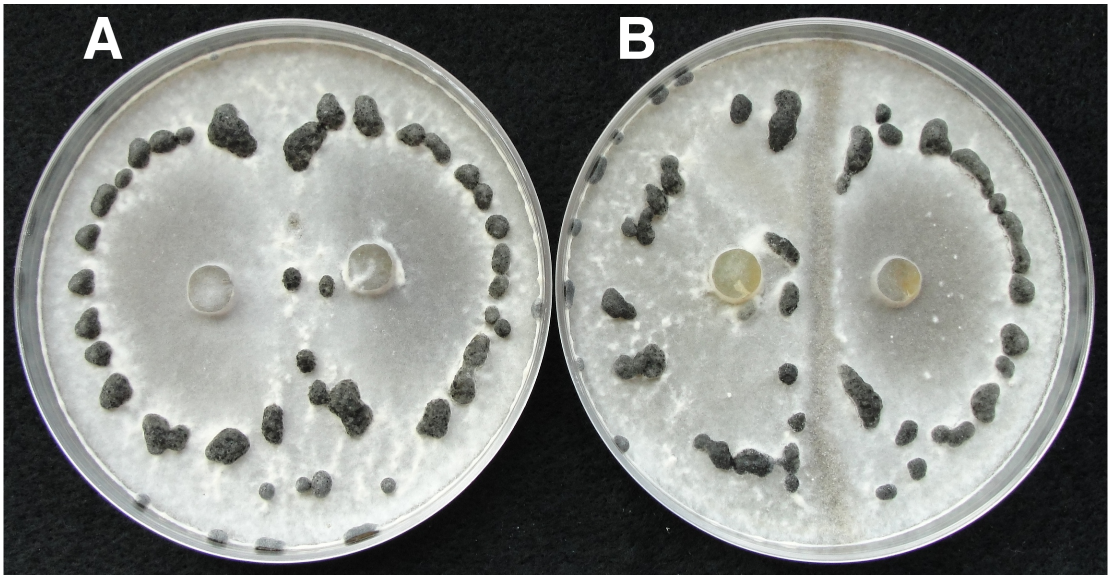

Figure S1. Example of MCG test plates showing (A) a compatible reaction with mycelia from two strains overgrowing each other and (B) an incompatible reaction with a barrage line of dead tissue forming between the two strains. Photo Credit: Rebecca Higgins.

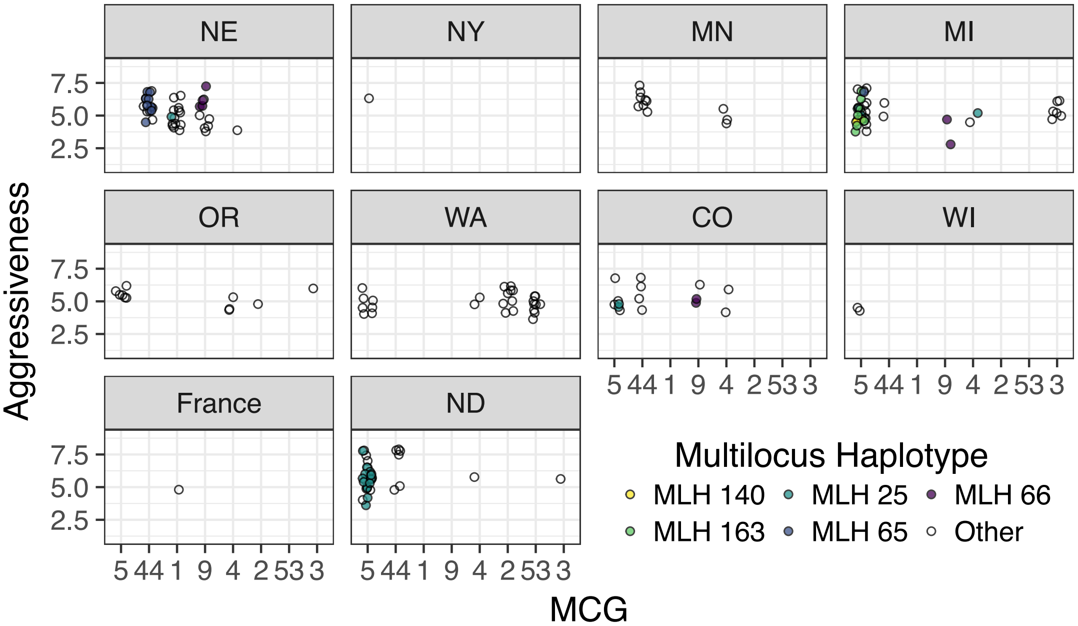

Figure S2. Strip plot of aggressiveness for the eight most abundant MCGs partitioned by region. Filled circles indicate one of the five most abundant MLHs and open circles indicate a MLH of lesser abundance.

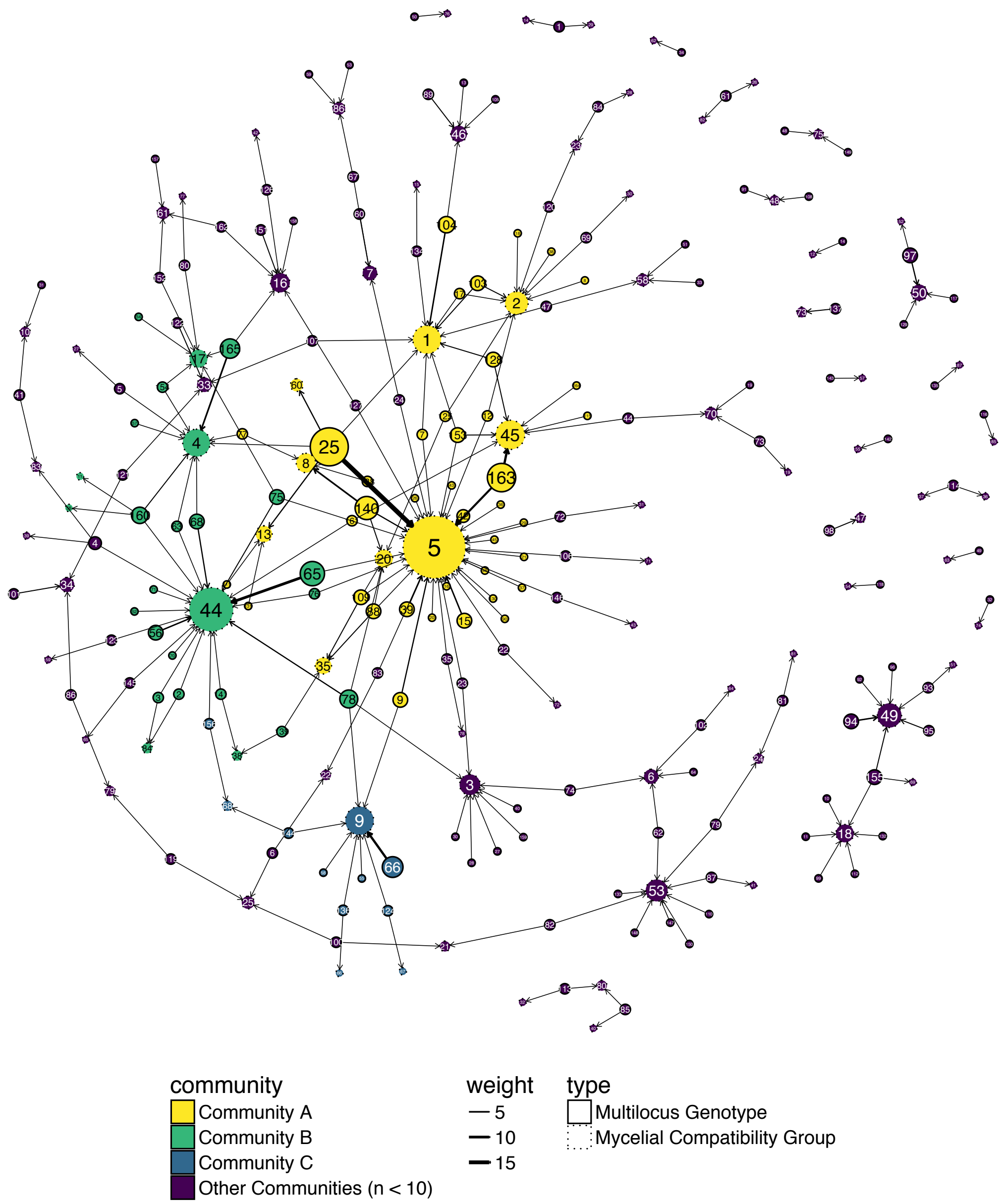

Figure S3. Graph showing complex associations between Mycelial Compatibility Groups (MCG) (dotted nodes) and Multilocus Haplotypes (MLH) (full nodes) where the number in each node represents the MLH/MCG assignment. Node size reflect the number of samples represented by each node (circle). Edges (arrows) point from MLH to MCG where the weight (thickness) of the edge represents the number of samples shared. Node color represents the community assignment based on the walktrap algorithm with a maximum of four steps (Pons & Latapy, 2006). An interactive version of this network can be recreated using the code in the "Interactive visualizations" section of the mlg-mcg.md file in the supplementary information (Direct Link: `https://github.com/everhartlab/sclerotinia-366/blob/master/results/mlg-mcg.md#interactive-visualizations`) (Kamvar et al., 2017).

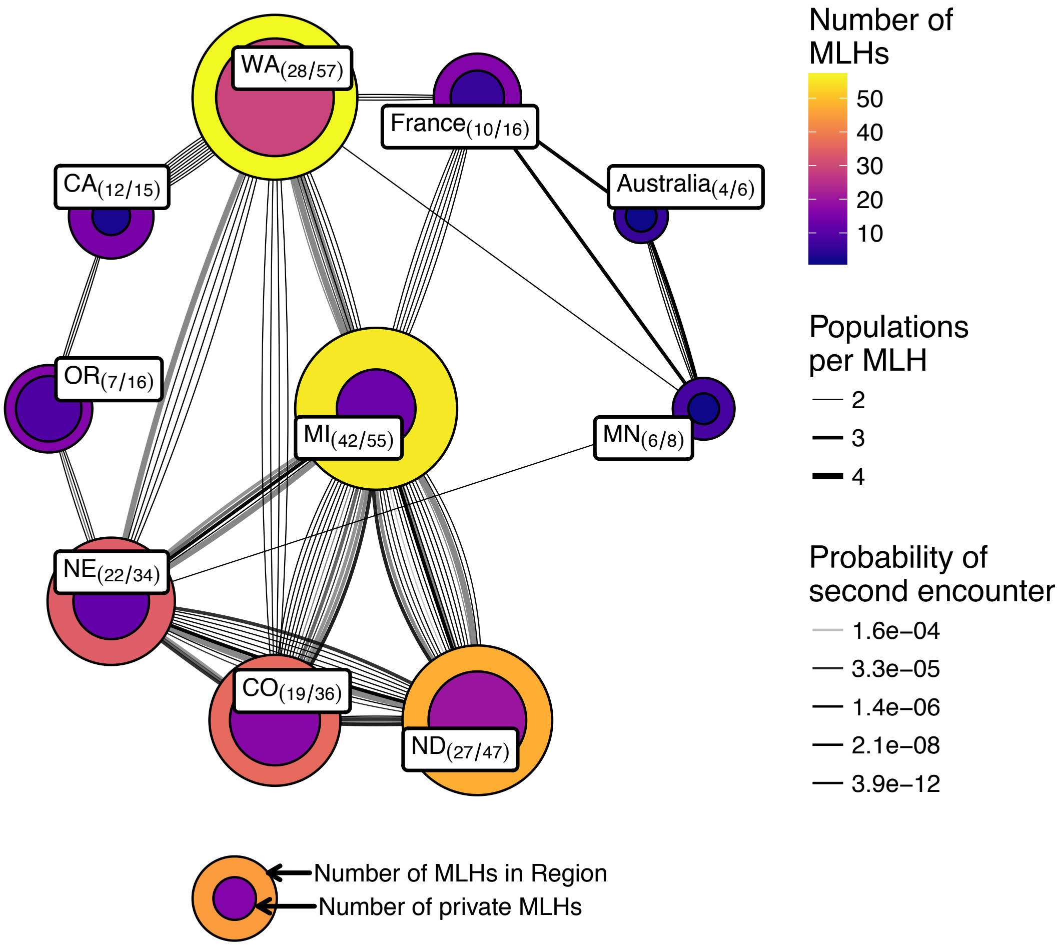

Figure S4. Network of populations (nodes/circles) and their shared multilocus haplotypes (MLH) (edges/lines) haplotyped over 16 loci. Each node is labeled with **name (number of MLHs shared/number of MLHs total).** The shade and area of the nodes are proportional to the number of unique MLHs within the node and the inner nodes are proportional to the number of private MLHs to the region (bottom legend). Each edge represents a single MLH where its thickness represents the number of populations that share the MLH and the shade represents the value of P_{sex}, or the probability of encountering that MLH from two independent meiotic events.

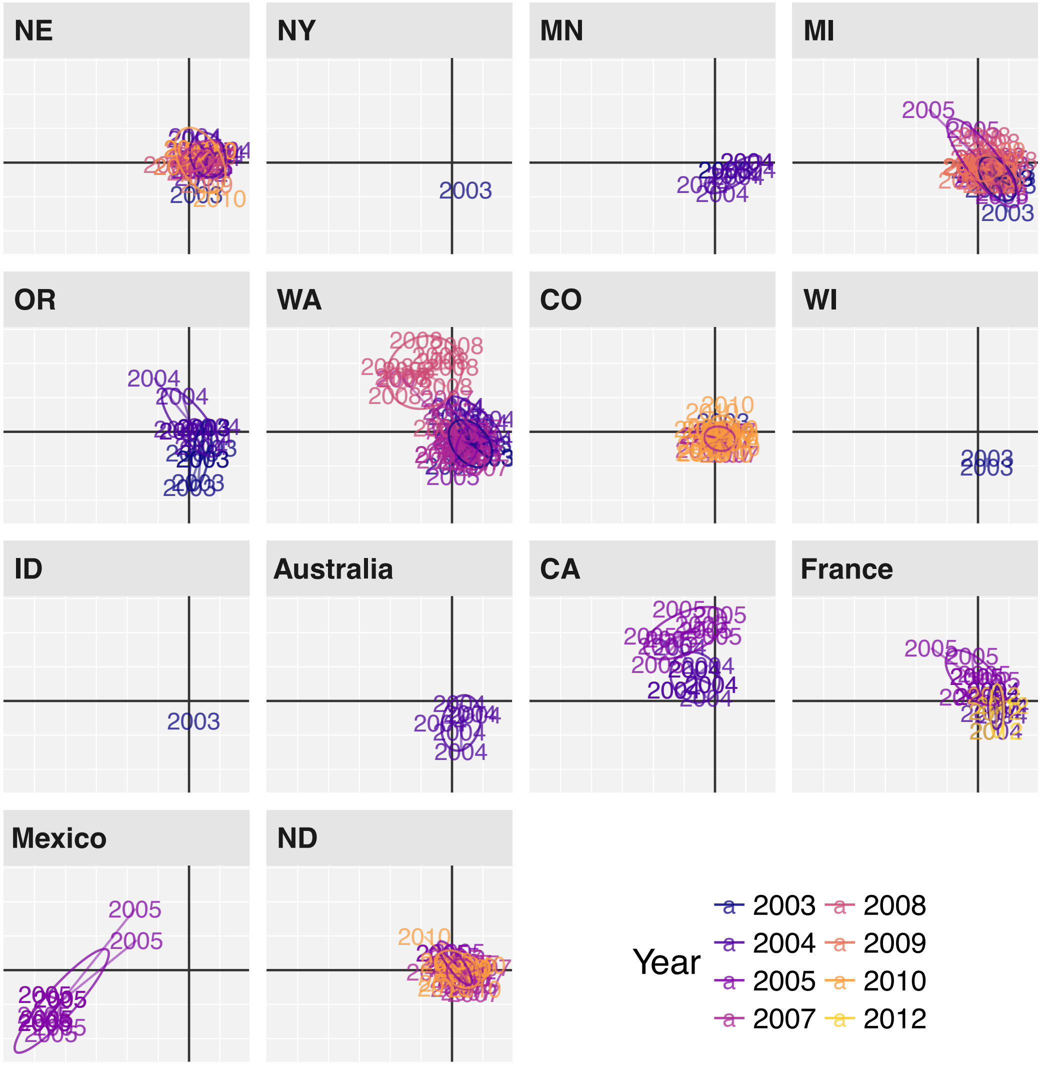

Figure S5. Scatter plot of Discriminant Analysis of Principal Components on Regions and Years showing temporal variation across all Regions. Points (text labels) represent observed individuals connected to the population centroids with ellipses representing a 66% confidence interval for a normal distribution. The center of each component is represented as black grid lines.
